# A privacy-preserving platform oriented medical healthcare and its application in identifying patients with candidemia

**DOI:** 10.1038/s41598-024-66596-8

**Published:** 2024-07-06

**Authors:** Siyi Yuan, Song Xu, Xiao Lu, Xiangyu Chen, Yao Wang, Renyi Bao, Yunbo Sun, Xiongjian Xiao, Longxiang Su, Yun Long, Linfeng Li, Huaiwu He

**Affiliations:** 1 Peking Union Medical College Hospital (CAMS), Beijing, China; 2 Yidu Cloud Technology Company Ltd., Beijing, China; 3 Peking Union Medical College Graduate School, Beijing, China; 4 Department of Intensive Care Unit, Affiliated Hospital of Qingdao University, Qingdao, China; 5 Department of Respiratory and Critical Care Medicine, First Affiliated Hospital of Fujian Medical University, Fuzhou, China; 6 Department of Biomedical Engineering, School of Life Science, Beijing Institute of Technology, Beijing, 100081 China

**Keywords:** Federated learning, Candidemia, Privacy-preserving, Predictive modeling, Feature selection, Diseases, Health care, Engineering, Mathematics and computing

## Abstract

Federated learning (FL) has emerged as a significant method for developing machine learning models across multiple devices without centralized data collection. Candidemia, a critical but rare disease in ICUs, poses challenges in early detection and treatment. The goal of this study is to develop a privacy-preserving federated learning framework for predicting candidemia in ICU patients. This approach aims to enhance the accuracy of antifungal drug prescriptions and patient outcomes. This study involved the creation of four predictive FL models for candidemia using data from ICU patients across three hospitals in China. The models were designed to prioritize patient privacy while aggregating learnings across different sites. A unique ensemble feature selection strategy was implemented, combining the strengths of XGBoost’s feature importance and statistical test *p* values. This strategy aimed to optimize the selection of relevant features for accurate predictions. The federated learning models demonstrated significant improvements over locally trained models, with a 9% increase in the area under the curve (AUC) and a 24% rise in true positive ratio (TPR). Notably, the FL models excelled in the combined TPR + TNR metric, which is critical for feature selection in candidemia prediction. The ensemble feature selection method proved more efficient than previous approaches, achieving comparable performance. The study successfully developed a set of federated learning models that significantly enhance the prediction of candidemia in ICU patients. By leveraging a novel feature selection method and maintaining patient privacy, the models provide a robust framework for improved clinical decision-making in the treatment of candidemia.

## Introduction

Artificial intelligence (AI) has shown a promising future in various fields such as computer vision^1^, natural language processing^2^, speech recognition^3^, and intelligent healthcare^4^. However, higher performance machine learning (ML) models require larger and diverse datasets which conflict with information protection since the leakage of personal information caused severe problems^5^. To protect privacy information, Europe released the General Data Protection Regulation^6^ (GDPR) in 2018 and China has published the Personal Information Protection Law^7^. In the USA, the Health Insurance Portability and Accountability Act^8^ was enacted to protect and guide the usage of patients’ medical information. To meet the requirements of legal law registration as well as making the best of datasets located across devices, Google proposed the federated learning (FL) as a possible solution in 2016^9^. In typical FL scenarios, only the updated model weights instead of original data samples are transferred between participant nodes. In a FL program, all participants incorporate to establish a global model derived from traditional ML models. The FL model is deemed to own comparative performance to a centralized model trained by collecting datasets into a center^10^. A FL task is usually categorized into three classes based on how datasets are distributed^11^: horizontally federated learning sharing the same feature space, vertical federated learning sharing same IDs and transfer federated learning. In addition, multiple cryptographic protocols [Secure Multiple Party Computation (MPC)^12^, homomorphic encryption (HE)^13^ and Differential Privacy (DP)^14^] can be integrated to enhance the FL framework.

Researchers and practitioners are gathering to promote the advancement of FL. Cheng et al.^15^ has established the lossless security boosting tree, Giacomelli et al.^16^ constructed the random forest at each silo and gathering the forests to make inference. And gigantic efforts are devoted to reduce the communication burden, heterogeneity of datasets and higher performance in FL. Currently, there exists several industrial FL frameworks such as Tensorflow Federated (TFF)^17^, Federated AI Technology Enabler (FATE), Paddle Federated Learning (PFL)^13^ and PySyft^18^ etc. All the above systems support both simulation and federated mode coupled multiple algorithms. However, to the best of our knowledge, there’s still not a FL system that oriented medical analysis which asks for specific requirements such as feature selection, factor interpretability and statistical tests. The comparisons about different FL algorithms have not been discussed further.

In this study, we propose a privacy-preserving platform YiDuManda oriented medical field and distinguish patients with candidemia. Candidemia is life-threatening infection which has become a leading cause of death among ICU patients. In our previous study, we have conducted a model to distinguish patients with candidemia in a center^19^. So far as we know, only a few federated learning models have applied in clinical medicine field^20–24^, and mainly focuses on algorithm development and adaption to medical application. In this paper, we take an overall comparison among four FL algorithms in classifying patients with candidemia. We assume that datasets across silos share the same features. We have implemented XGBoost, logistic regression (LR), random forest (RF) and support vector machine (SVM) in the federation scenario on YiDuManda. The FL XGBoost is implemented based on multi-party sorting protocols^25^ and achieves similar performance as the centralized non-privacy-preserving method. We developed the FL LR and SVM with the same strategy federated averaging algorithm^9^ (FedAvg). The SVM is derived from sklearn tool kit^26^ on Python platform and calibrated on the parametric sigmoid formula proposed by Platt^27^. And the FL RF is carried out in an assemble way that each silo constructs one RF separately during the training process. All the above methods have three modes: federated mode, local mode and global mode. And the trained models are FL model, locally model and centralized model respectively. And we devoted to make predictions about patients with candidemia in ICU.

To get an optimal subset of features related to candidemia, we proposed an ensemble feature selection method that collaborates the feature importance of XGBoost^28^ model and the *p* values between features and label.

## Methods

### Data sources and patients

We collected the ICU patients greater than 14 years old from three hospitals: Peking Union Medical College Hospital (Pumch), The Affiliated Hospital of Qingdao University (Qyfy), The First Affiliated Hospital of Fujian Medical University (Fyyy). This study has been approved by the ethics committee of the three hospital (SK693, QYFYKYLL 601311920, FMU244). Patients who were admitted to the above target hospitals and had new-onset systemic inflammatory response syndrome (SIRS) from 2013 to 2017 were selected as the subjects of the study. New-onset SIRS needed to meet the following criteria: (1) SIRS occurred in the ICU; (2) blood culture was obtained during the course of SIRS; (3) no previous SIRS within 24 h. This content has been described in detail in our previous research. The exclusion criteria includes: (1) age < 14 years old; (2) SIRS occurred out of ICU; (3) no blood cultures obtained during SIRS. Positive patients with candidemia were diagnosed by the positive blood culture results which show the presence of Candida species after ICU reception.

Upon conducting a thorough review of prior research, a comprehensive set of 22 risk factors exhibiting strong clinical relevance to candidemia was identified. These factors encompass four distinct categories: basic patient information, associated comorbidities, laboratory blood test results, and treatment histories. The datasets obtained from the three participating hospitals share a uniform feature space, ensuring consistency in the scope of variables considered for analysis. This meticulous selection and harmonization of risk factors facilitate a robust and comprehensive evaluation of the predictors of candidemia.

### YiDuManda framework

YiDuManda is a sophisticated framework designed to enable privacy-preserving data mining across various data silos, integrating three key services: Management Service, Computation-Engine Service, and Network Communication Service. The Management Service oversees the entire lifecycle of a data mining task, including initiation and computing resource allocation, ensuring efficient task progression. The Computation-Engine Service provides fundamental computational capabilities, supporting secure multi-party computation, differential privacy, and homomorphic encryption, crucial for data integrity and confidentiality. Inter-service communication within YiDuManda is facilitated through the gRPC protocol, known for its strong performance^29^ and security features^30^. The platform also offers high-level Python interfaces, enhancing its scalability and usability for data scientists. Complementing this architecture, YiDuManda includes several user-friendly modules: a Machine Learning Algorithms Module with various methods for classification, regression, and ranking; a Statistics Module for comprehensive data overviews and trend analysis; and a Feature Engineering Module focusing on feature standardization, selection, and transformation. Collectively, YiDuManda stands out as an all-encompassing, versatile framework for privacy-sensitive data mining, equipped with a broad spectrum of tools for advanced data analysis and machine learning applications.

### Model development and performance evaluation

In our experimental design, the dataset was partitionally randomized into a training set and a testing set at each silo, adhering to an 8:2 split ratio, respectively. The testing sets from the three distinct silos were then collectively aggregated on a centralized server. Throughout the training phase, the federated learning (FL) model weights underwent transfer amongst participating entities. Each local model was exclusively trained on its respective dataset, while the centralized model training was conducted using a selection of three training datasets from the participating hospitals. The efficacy of all models was rigorously evaluated using the testing sets derived from these hospitals. For performance assessment, we employed several key metrics: the area under the curve (AUC) of the receiver operating characteristics (ROC) curve, the true positive rate (TPR), and the true negative rate (TNR). To ensure the robustness of our findings, this experimental procedure was replicated 15 times. In terms of feature selection, we adopted the "TPR + TNR" criterion, as proposed by Yuan et al.^19^. This criterion was operationalized through a hybrid linear searching algorithm, which was utilized to identify the configuration yielding the highest combined TPR and TNR scores. This methodological approach was instrumental in enhancing the precision of our feature selection process and choose the highest one by a hybrid linear searching algorithm.

### Privacy-preserving boosting tree

**Figure 1:**
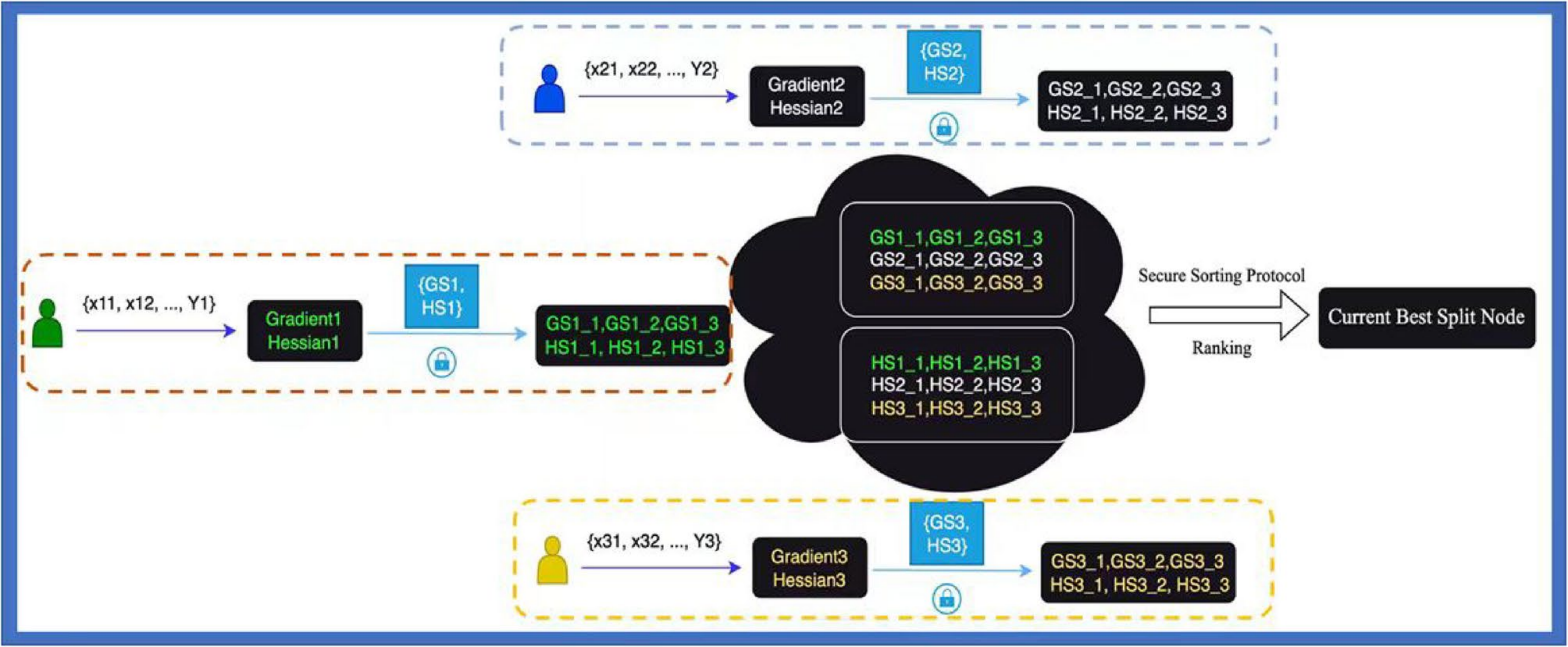
Best-split calculation for XGBoost.

In the realm of computational engineering, the boosting tree methodology has been recognized as a highly effective and versatile tool, as evidenced by its application in various domains^31,32^. Particularly, the implementation of secure boosting trees has been adapted to different data partitioning strategies, including vertically partitioned data^33^ and horizontally partitioned data^15^. In our research, we have advanced the secure boosting tree approach by integrating secure multi-party computation sorting^25^. We set a secure sorting network on shares and obtain the positions of values in each feature. The positions are declared to every participant. As shown by Fig. 1, the sum of local gradients and hessians are calculated coupled with pairwise masking at each silo. Each participant sends the two masked sums to the manager and the best split feature can be found. The position of best feature as well as the participant whom the position belongs to are kept in the tree structure. This is necessary to make predictions since the model has no idea of the threshold at each node. The whole process is depicted in Fig. 1.

### Privacy-preserving SVM and LR

**Figure 2:**
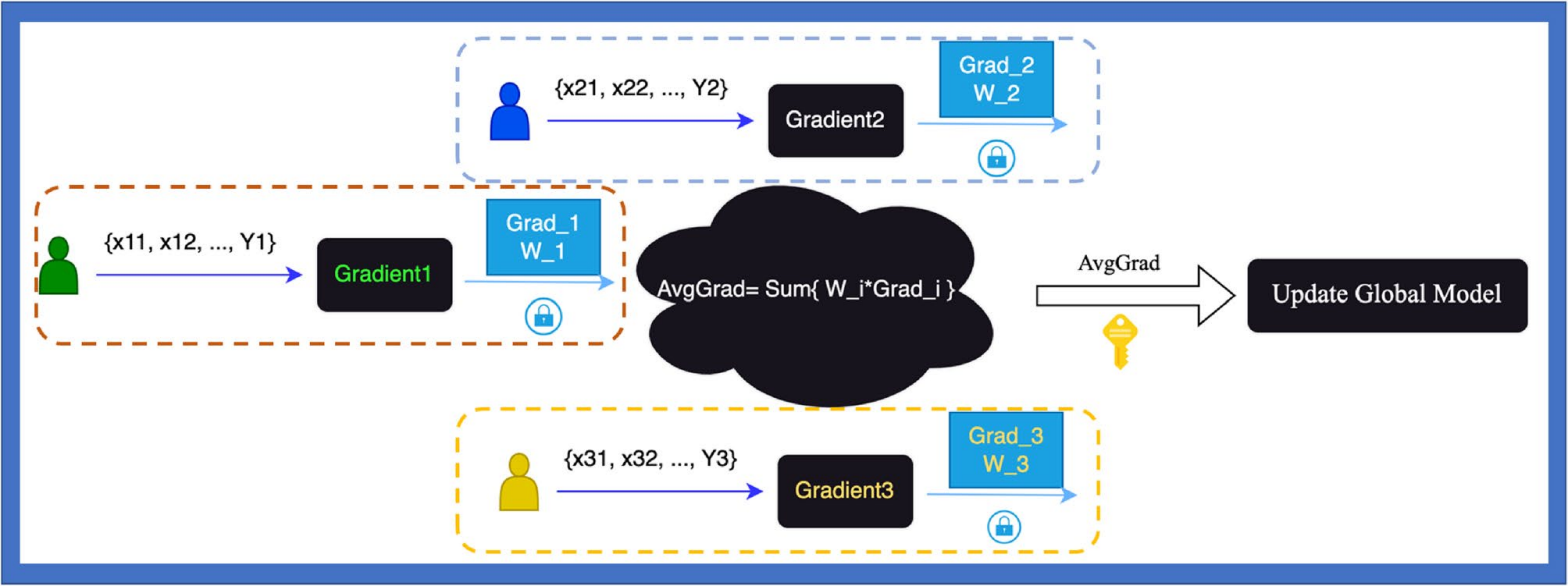
Flow of secure logistic regression and SVM.

In the domain of supervised machine learning classification, both Logistic Regression (LR) and Linear Support Vector Machine (SVM) are recognized for their strong interpretative capabilities. McMahan et al.^9^ introduced the Federated Averaging Algorithm (FedAvg), a groundbreaking approach for implementing federated neural networks. This algorithm allows for each client in the network to perform multiple iterations of local updates prior to synchronizing with the central server, a methodology we adopted for federated LR in our system. The neighbors can be acquired by numbering and sorting the participants. And the whole process of secure progress is show in Fiture2. To secure the averaging process in the server, we made the pairwise masking by applying Diffie-Hellman key exchanging protocol^34^. To get the probabilistic outputs of the federated SVM, we have adopted the parametric sigmoid formula suggested by Platt^27^ and calculated the optimized parameters with FEDSGD^9^. And the constructing workflow is depicted in Fig. 2.

### Privacy-preserving RF

Random Forest (RF) is an ensemble classifier renowned for its ability to construct numerous independent decision trees and derive predictions by aggregating the outcomes from each tree. In our research, we have developed a privacy-preserving RF model, employing the methodology advocated by Vaidya^35^ and utilizing the sklearn toolkit within a Python 3.6 environment. This approach assumes that the model’s structure, while sensitive, does not carry the same level of vulnerability as raw data, allowing for its controlled dissemination to select users. Looking ahead, our aim is to enhance the security framework of RF, focusing on minimizing the risk of information leakage and further fortifying the model’s privacy-preserving capabilities.

### Feature engineering

Within statistical analysis, two prevalent hypothesis tests are the chi-square test and the Student’s t-test. The chi-square test is primarily utilized to assess and compute the associations between two categorical variables, while the Student’s t-test is employed to compare the differences in continuous variables across different groups. Typically, the correlation coefficient between variables is indicative of their mutual dependence. In the realm of feature selection, the objective often involves identifying a smaller yet more significant subset of variables. In a previous study, Yuan et al.^19^ successfully identified risk factors associated with candidemia using XGBoost exclusively. Building upon this, we have developed a novel hybrid feature selection method tailored for federated settings. This method synergistically combines hypothesis testing and correlation coefficient analysis, thereby enhancing the robustness and relevance of the selected features. In the study, we have computed the Pearson Coefficient between x and y like this: calculate the global mean for each variable $\overline{x } and \overline{y }$ locally; then get the sum of (x-$\overline{x }$), (y-$\overline{y }$), (x-$\overline{x }$)^2^ and (y-$\overline{y }$)^2^; finally, we can calculate the Pearson Coefficient value.

The hybrid approach was carried out by combining statistical analysis and feature importance of XGBoost. The results are subset-A and subset-B selected by two methods. The Subset-B was obtained as the approach proposed by Yuan S. The statistical selection approach is as follows: (1) calculating the *p* values for each variable and sorting in an ascending order; (2) eliminate the feature with the largest *p* value and constructing XGBoost on the left features; (3) repeat the second step until no more features can be delete; (4) assigning subset-A as the subset that reaches the highest level of “TPR + TNR; (5) calculate the intersection of subset-A and subset-B and assign the result as subset-C; (6) calculate the coefficient correlations of features in subset-C; (7) remove the redundant variables which coefficient is more than threshold.

### Ethical approval

The ethics was approved by the ethics committe of Peking Union Medical College Hospital (Reference Number: S-K693). All patients data has been anonymized before sharing among researchers. And Informed consent was obtained from all the participants involved in the study. The experiment was conducted in adherence to the World Medical Association Declaration of Helsinki Ethical Principles for Medical Research Involving Human Subjects.

### Consent for publication

All listed authors consented to the submission and all data were used with the consent of the person generating the data.

## Results

### Basic characteristics

**Figure 3:**
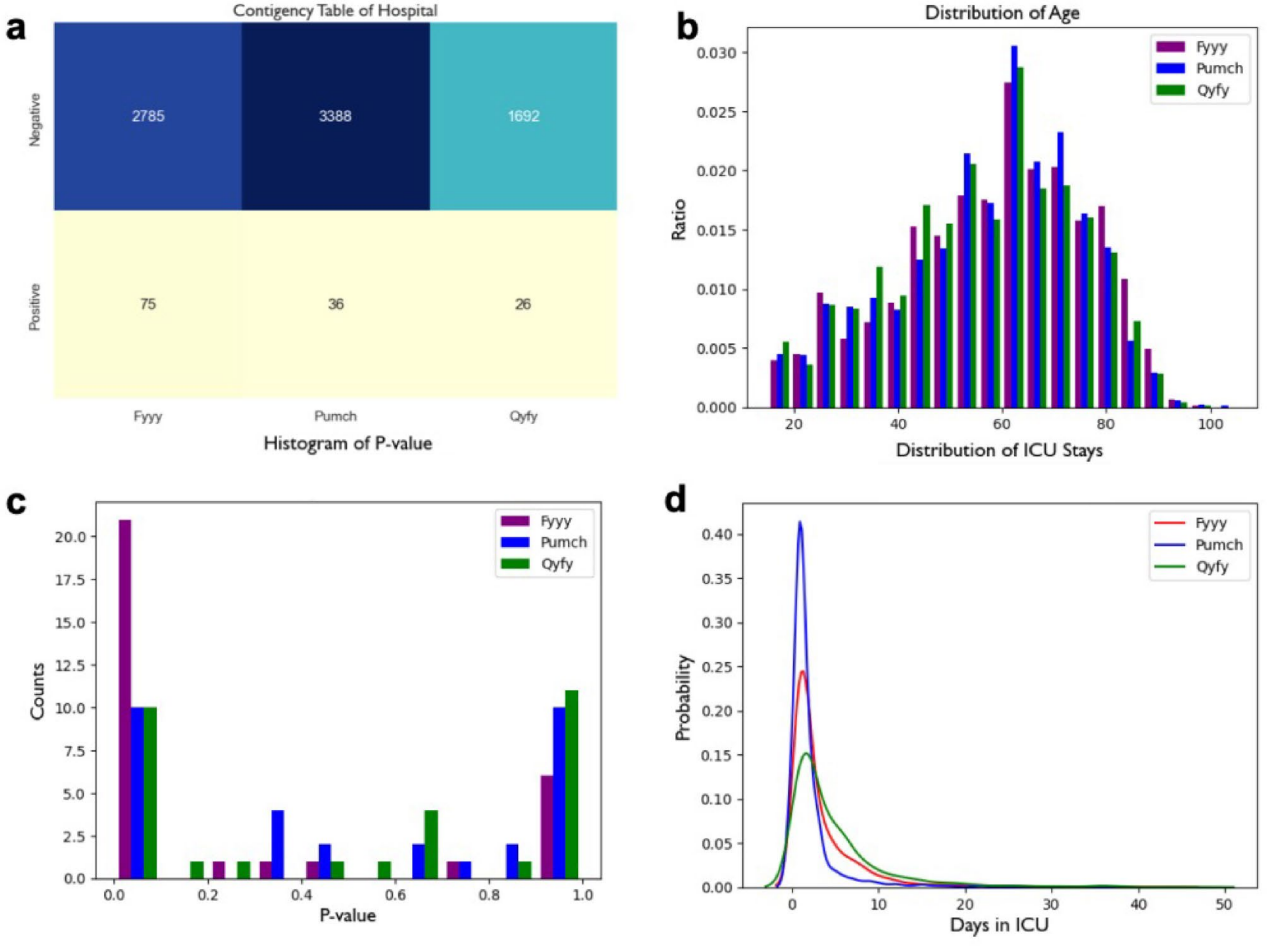
Demographics of datasets from three hospitals. (**a**) contingency table of positives and negative in three hospitals; (**b**) probability distribution histogram of ages in three hospitals; (**c**) histogram of *p* values in three hospitals; d, distribution of ICU Days in three hospitals.

A total of 8002 ICU patients with 22 features are enrolled from three hospitals Fyyy (n = 2860), Pumch (n = 3424) and Qyfy (n = 1718). And the ratios of patients with blood culture positive for candidaemia are 0.62% (Fyyy), 1.05% (Pumch) and 1.51% (Qyfy). There is a significant difference in the samples distribution among three hospitals (*p* < 0.001, chi-square test). Of all the selected features, two types of variables are included: continuous ones and categorical ones. The frequency distribution of patients’ age is shown in Fig. 3b (*p* = 0.995, Kruskal–Wallis H-test). And we calculated the *p* values between variables and labels are calculated through chi-square test and the histogram is plotted in Fig. 3c (*p* = 0.338). The density curve of ICU stays is shown in Fig. 3d **(***p* < 0.001, chi-square test). The Fig. 3 implies that there is similarity as well as diversity in the characteristic among the patients.

### Model results

**Figure 4:**
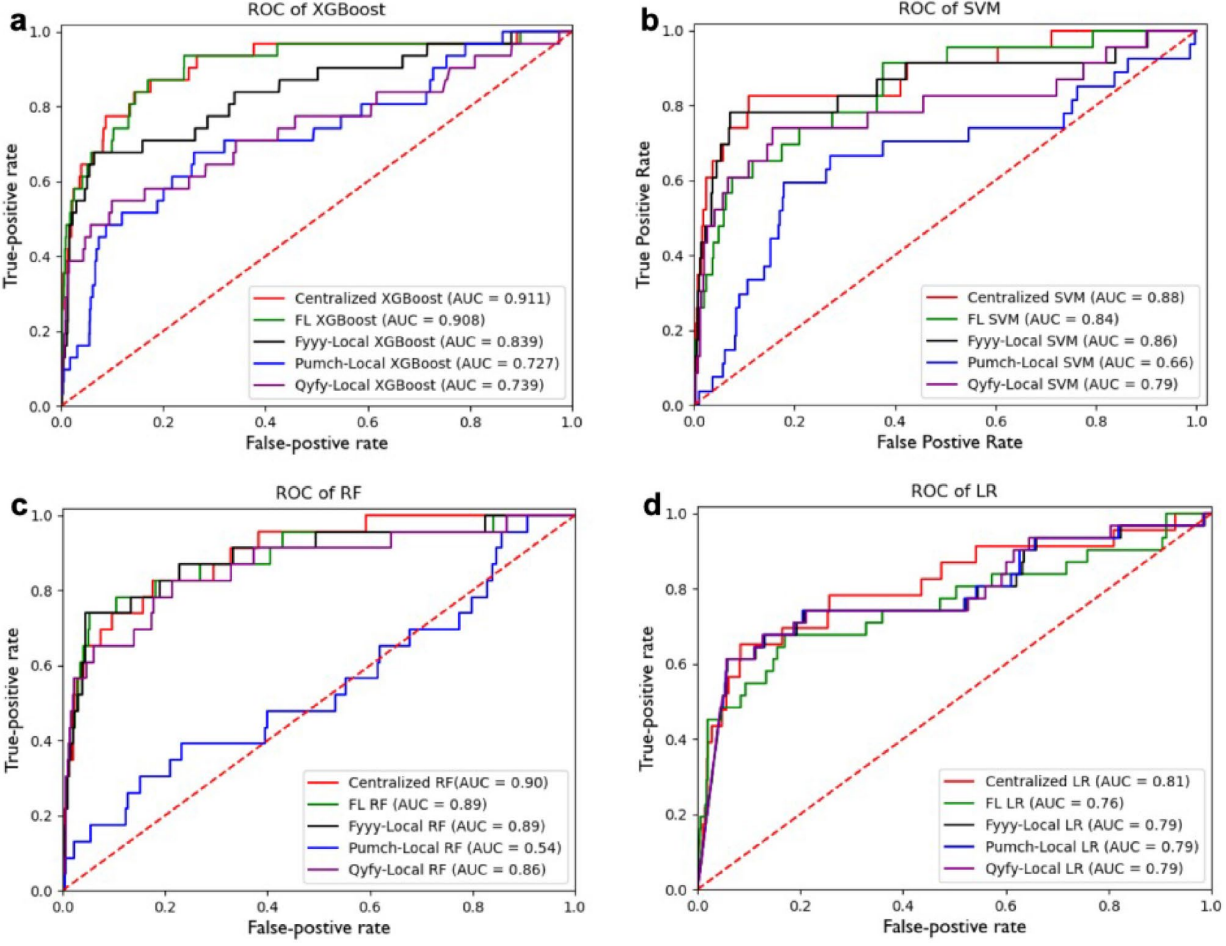
Comparisons of centralized, FL and locally trained models with different algorithms. (**a**) ROC of XGBoost algorithms; (**b**) ROC of SVM algorithms; (**c**) ROC of RF algorithms; (**d**) ROC of LR algorithms.

We have made a thorough comparisons on four machine learning algorithms in distinguishing patients with candidaemia. The datasets from three hospitals are split into training sets and testing sets on the ratio 8:2 respectively. For each algorithm, we constructed models in three types: centralized model, FL model and locally model. The best ROCs of each model were shown in Fig. 4 and the averaging AUCs were entailed in online Appendix Table 1.

The Fig. 4 displayed the best ROCs for each algorithm: Fig. 4a-XGBoost, b-SVM, c-RF, and d-LR. The results showed that the centralized models perform best with an average AUC of 0.875. Specially, the centralized XGBoost resulted in AUC-0.911 that approaches the previous study^19^ which captures SMOTE mechanism. The best FL models achieved an average AUC of 0.849 and the average performance of best local models 0.773. In summary of online Appendix Table 1, the FL models except LR generally gained improvements in “mean-AUC”, “Mean-TPR”, “TPR + TNR” than local models. To be specific, the mean-AUC of FL models are: 0.821 in XGBoost, 0.771 in SVM, 0.817 in RF and 0.724 in LR. The characteristic of “TPR + TNR” is: 1.519 in XGBoost, 1.451 in SVM, 1.526 in RF and 1.358 in LR. The robustness of experiment is validated by repeating 15 runs on different randomly splits.

**Figure 5:**
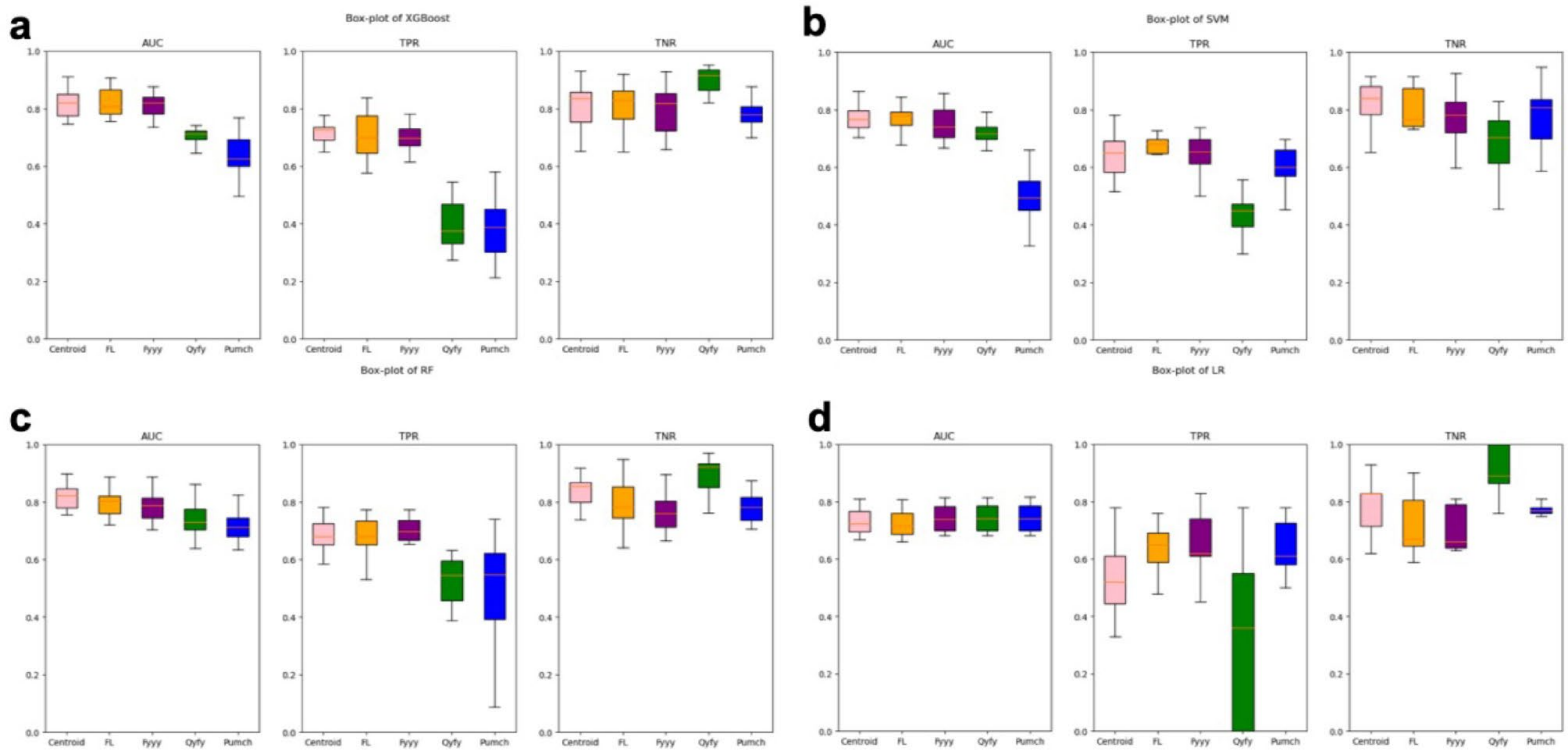
Boxplot of models count on AUC, TPR, TNR. (**a**) the boxplot about AUC, TPR and TNR of XGBoost; (**b**) the boxplot about AUC, TPR and TNR of SVM; (**c**) the boxplot about AUC, TPR and TNR of RF; (**d**) the boxplot about AUC, TPR and TNR of LR.

The detailed boxplot about the comparisons of four algorithms was illustrated in Fig. 5. It’s clearly that the centralized models and FL models both possess better stabilities and generalities than local ones. And the FL models can achieve comparable achievements as well as the centralized ones. What’s more, locally models have acquired diverse performance on testing set. It may come from the heterogeneity of datasets from three hospitals. In detail, local models can achieve relatively better states on a single factor but dropped on others which restricts its generality.

### Feature selection

A typical process of feature selection has been proposed by combining feature importance and statistical analysis in online Appendix Fig. 1. The *p* values of features correspond to the label and sorted in an ascending order in Table 1. Naturally, a larger *p* value indicates a less important factor in discrimination. The Table 2 showed the most suitable subset has been acquired after eliminating the “Severe Sepsis”. The number of features in subset-A is 16. The subset-B was acquired by the strategy proposed by Yuan S. In the method, feature importance is obtained by constructing XGBoost model and then exclude the least one in a greedy manner. The process ends until the best performance has been reached. However, it has been commonly accepted that a single selection method is biased in feature selection. And a hybrid approach may benefit from different methods. By taking the intersection of subset-A and subset-B, we can obtain a smaller number of subset but contains the most import variables and the result features are listed in Table 3. Lastly, the coefficient correlations between variables was considered to filter redundant ones (threshold = 0.3) that seems less important in feature importance. And the final subset of features is shown in Table 4 and contains 12 variables. The performance of XGBoost training on the final subset is AUC-0.92, TPR-0.84, TNR-0.88 and cut-off value is 0.029. By taking the hybrid approach, we have obtained similar performance as previous study on a smaller number of features.

**Table 1:** The federated *p* values of features.

Serial number	Risk factors	*p* value(federation)
1	Colonization	< 0.0001
2	Days of central venous catheter	< 0.0001
3	Days of mechanical ventilation	< 0.0001
4	History of fungal infection	< 0.0001
5	BDG positive	< 0.0001
6	Days of total parenteral nutrition	< 0.0001
7	Length of hospital stay	< 0.0001
8	Abdominal surgery	< 0.0001
9	Days of renal replacement therapy	< 0.0001
10	Length of ICU stay	< 0.0001
11	LYM^#^ < 1 × 10^9^/L	< 0.0001
12	NEUT^#^ < 1.5 × 10^9^/L	< 0.0001
13	Corticosteroid therapy or immunosuppressive use	0.007
14	Broad-spectrum antibiotic use	0.0136
15	Malignant tumor	0.04
16	Acute kidney injury	0.081
17	Severe Sepsis	0.178
18	Diabetes	0.308
19	Sex	0.769
20	Age	0.859
21	Pancreatitis	1.0
22	Chemotherapy drug use	1.0

**Table 2:** The performance of feature subset by statistical analysis.

Serial number	Eliminating feature	AUC	TPR	TNR	Thres
1	Colonization	–	-	–	–
2	Days of central venous catheter	–	–	–	–
3	Days of mechanical ventilation	–	–	–	–
4	History of fungal infection	–	–	–	–
5	BDG positive	–	–	–	–
6	Days of total parenteral nutrition	–	–	–	–
7	Length of hospital stay	–	–	–	–
8	Abdominal surgery	–	–	–	–
9	Days of renal replacement therapy	0.91	0.61	0.91	0.044
10	Length of ICU stay	0.920	0.74	0.81	0.02
11	LYM^#^ < 1 × 10^9^/L	0.951	0.71	0.9	0.037
12	NEUT^#^ < 1.5 × 10^9^/L	0.960	0.74	0.89	0.031
13	Corticosteroid therapy or immunosuppressive use	0.95	0.77	0.89	0.033
14	Broad-spectrum antibiotic use	0.963	0.61	0.94	0.041
15	Malignant tumor	0.961	0.74	0.91	0.041
16	Acute kidney injury	0.967	0.74	0.90	0.03
17	Severe sepsis	0.954	0.81	0.92	0.034
18	Diabetes	0.962	0.74	0.93	0.042
19	Sex	0.940	0.81	0.89	0.03
20	Age	0.943	0.77	0.9	0.032
21	Pancreatitis	0.975	0.61	0.95	0.053
22	Chemotherapy drug use	0.973	0.68	0.92	0.035

**Table 3:** Intersection of subset-A and subset-B.

Serial number	Risk factors
1	Colonization
2	Days of central venous catheter
3	Days of mechanical ventilation
4	History of fungal infection
5	BDG positive
6	Days of total parenteral nutrition
7	Length of hospital stay
8	Abdominal surgery
9	Days of renal replacement therapy
10	Length of ICU stay
11	LYM^#^ < 1 × 10^9^/L
12	NEUT^#^ < 1.5 × 10^9^/L
13	Corticosteroid therapy or immunosuppressive use
16	Acute kidney injury

**Table 4:** The final subset of feature selection.

Serial number	Risk factors
1	Colonization
3	Days of mechanical ventilation
4	History of fungal infection
5	BDG positive
6	Days of total parenteral nutrition
7	Length of hospital stay
9	Days of renal replacement therapy
10	Length of ICU stay
11	LYM^#^ < 1 × 10^9^/L
12	NEUT^#^ < 1.5 × 10^9^/L
13	Corticosteroid therapy or immunosuppressive use
16	Acute kidney injury

## Discussion

In this study, we introduce YiDuManda, a privacy-preserving framework comprising three core modules: a Machine Learning Module, a Statistics Module, and a Feature Engineering Module. This framework has been effectively applied in a real-world clinical setting for classifying patients with candidemia and identifying disease-relevant features. Within this framework, we have implemented four types of algorithms: XGBoost, SVM (Support Vector Machine), LR (Logistic Regression), and RF (Random Forest). The federated learning (FL) models within YiDuManda have demonstrated performance comparable to centralized models, exhibiting enhanced generality and improved performance compared to models trained solely on local datasets. This underscores the FL model’s capability to strike a balance between privacy protection and data utilization.

A comprehensive evaluation and comparison of various algorithms and models were conducted. And the detailed results of all models are shown in online Appendix Table 1. Although the FL XGBoost emerged as the most effective method, its construction and training processes are complex and time-consuming, primarily due to the integration of secure multi-party computation (MPC) sorting. Conversely, the FL RF model, while simpler in its construction, achieved performance closely matching that of XGBoost. It is important to note that no single model uniformly outperformed others across all metrics, highlighting the necessity of carefully selecting algorithms tailored to specific clinical contexts. This strategic selection is crucial for optimizing performance and applicability in real-world clinical scenarios.

The demographic analysis of our dataset revealed an imbalance in sample distribution between negative and positive labels, with notable discrepancies in positive patient ratios across the three participating hospitals. This observation underscores the need for diverse optimization strategies in real-world applications^36^. Enhancing the performance of federated learning (FL) models may be achieved through a retrospective analysis of misclassified samples.

In recent trends, there is growing interest in developing personalized models that amalgamate the strengths of both FL and local models. Our experimental observations indicated that the Fyyy-local model outperformed its counterparts. Efforts are ongoing in the field to scrutinize dataset quality and filter out suboptimal samples, our approach incorporated a hybrid feature selection strategy to streamline the number of features. We posited that relying on a singular feature selection method might lead to biased outcomes in the quest for the optimal solution. Interestingly, we observed a correlation in the ranking of features sorted by *p* values and their corresponding importance scores. This led us to identify and eliminate ‘Diabetes’—a feature that had previously been ranked highly. Additionally, we removed features exhibiting correlation coefficients exceeding 0.3 with dominant indicators, such as the duration of central venous catheter use and history of abdominal surgery.

This research has some limitations. First, the study recognizes the heterogeneity in sample distribution across hospitals, which could be due to differences in hospital practices, patient demographics, and underlying conditions. The findings’ generalizability is limited and future studies are required to validate these results in larger, more diverse populations. Second, the study notes the benefits of FL models but does not address potential trade-offs such as communication overhead, data heterogeneity, and model convergence issues. In addition, considering the limitations of the SMOTE method^37^, we did not employ SMOTE for handling data imbalance, which may have a certain impact on the model’s performance. These trade-offs need to be considered when deploying FL in real-world applications, particularly in sensitive domains like healthcare. Third, the hybrid feature selection approach lacks validation for the chosen thresholds and criteria. This study established a machine learning candidaemia prediction model that could be implemented in a computer program to realize real-time bedside assessment of the possibility of developing candidaemia. But its integration into real world still needs to be clarified. Furthermore, to improve the interpretability and stability of models, more methods are being developed and analyzed, such as the Bayesian Neural Networks. The method can predict the results but also the confidence of the results on small sample size.

Looking forward, our aim is to encompass a broader spectrum of medical statistical tests and incorporate survival analysis within an FL framework. The criticality of making informed decisions in medical practice cannot be overstated, particularly as clinicians often manage numerous cases daily. An integrated end-to-end FL platform, which encompasses both the training and inference stages, is a prospective avenue for exploration^38^. Moreover, evaluating and fine-tuning the parameters of FL models is essential for enhancing their practical utility in clinical settings.

## Conclusion

This study developed effective federated learning models for predicting candidemia in ICU patients, significantly outperforming traditional models. The approach combined data from three hospitals, emphasizing patient privacy and innovative feature selection strategies. The models showed a marked improvement in predictive accuracy, indicated by increased AUC and TPR metrics, providing a robust framework for enhanced clinical decision-making in treating candidemia.

### Supplementary Information

Supplementary Information.

## Data Availability

The datasets and codes in the current study are available from the corresponding author upon reasonable request.
