# Supplementary material for: A privacy-preserving platform oriented medical healthcare and its application in identifying patients with candidemia
Source: Sci Rep. 2024 Jul 6;14:15589. doi: 10.1038/s41598-024-66596-8 (PMC11227531; doi:10.1038/s41598-024-66596-8)
Supplement: Supplementary file 1 — Supplementary Information. [file 41598_2024_66596_MOESM1_ESM.docx]

**Appendix**

Append Table 1. Performance of all models

| **Algorithms** | **Mean-AUC** | **Mean-TPR** | **Mean-TNR** | **TPR+TNR** |
| --- | --- | --- | --- | --- |
| Centralized XGBoost | **0.819** | **0.716** | 0.802 | **1.518** |
| FL XGBoost | **0.821** | **0.710** | 0.809 | **1.519** |
| Fyyy XGBoost | 0.810 | 0.697 | 0.793 | 1.49 |
| Pumch XGBoost | 0.640 | 0.394 | 0.785 | 1.179 |
| Qyfy XGBoost | 0.705 | 0.408 | 0.902 | 1.31 |
| Centralized SVM | 0.776 | 0.644 | 0.825 | 1.469 |
| FL SVM | **0.771** | **0.647** | **0.804** | **1.451** |
| Fyyy SVM | 0.754 | 0.646 | 0.775 | 1.421 |
| Pumch SVM | 0.495 | 0.432 | 0.684 | 1.116 |
| Qyfy SVM | 0.721 | 0.603 | 0.776 | 1.379 |
| Centralized RF | **0.817** | **0.699** | 0.827 | **1.526** |
| FL RF | 0.796 | 0.683 | 0.8 | 1.483 |
| Fyyy RF | 0.787 | 0.690 | 0.765 | 1.455 |
| Pumch RF | 0.708 | 0.490 | **0.894** | 1.384 |
| Qyfy RF | 0.737 | 0.522 | 0.784 | 1.306 |
| Centralized LR | 0.730 | 0.538 | 0.781 | 1.319 |
| FL LR | 0.724 | 0.639 | 0.719 | 1.358 |
| Fyyy LR | 0.743 | 0.664 | 0.703 | 1.367 |
| Pumch LR | 0.744 | 0.643 | 0.772 | 1.415 |
| Qyfy LR | 0.743 | 0.295 | **0.917** | 1.212 |


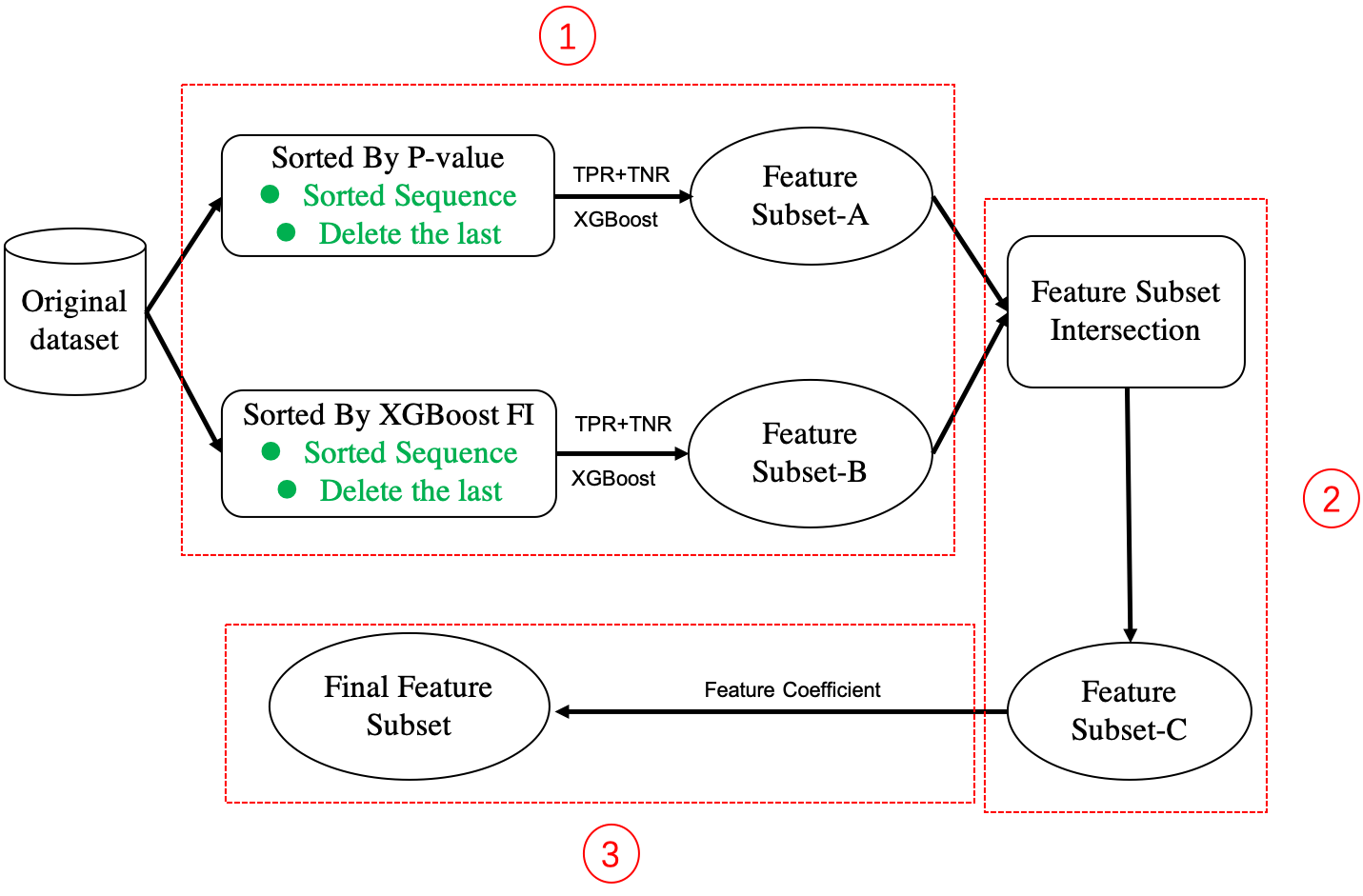


Append Figure 1. The workflow of feature selection
